# Obesity and metabolic abnormalities as risks of alcoholic fatty liver in men: NAGALA study

**DOI:** 10.1186/s12876-021-01893-4

**Published:** 2021-08-09

**Authors:** Yuta Yoshimura, Masahide Hamaguchi, Yoshitaka Hashimoto, Takuro Okamura, Naoko Nakanishi, Akihiro Obora, Takao Kojima, Michiaki Fukui

**Affiliations:** 1 Department of Endocrinology and Metabolism, Graduate School of Medical Science, Kyoto Prefectural University of Medicine, 465, Kajii-cho, Kawaramachi-Hirokoji, Kamigyo-ku, Kyoto, 602-8566 Japan; 2 Department of Gastroenterology, Asahi University Hospital, Gifu, Japan

**Keywords:** Alcohol-related liver disease, Fatty liver, Obesity, Dyslipidemia, Metabolic syndrome

## Abstract

**Background:**

Hepatic steatosis has a pivotal role in the development of chronic liver diseases, even in alcohol-related liver disease. Alcoholic fatty liver disease is an important phenotype among alcohol-related liver diseases. While metabolic syndrome is a dominant risk factor of incident nonalcoholic fatty liver disease, the role of metabolic syndrome in alcoholic fatty liver disease has not been clarified yet.

**Methods:**

A retrospective cohort study was performed at a health check-up center in Japan. Subjects consisted of male participants without fatty liver who consumed ethanol of 420 g/week or higher. Adjusted hazard ratios and 95% confidence intervals at the baseline examinations for incident alcoholic fatty liver disease were estimated using Cox model.

**Results:**

A total of 640 participants were included in this study. During 3.91 years (IQR 1.63–7.09) of follow-up, 168 new cases of alcoholic fatty liver disease developed (49.1 cases per 1000 persons per year). After adjustment for age, smoking status, alcohol consumption, the hazard ratio for a 1 kg/m^2^ increase in body mass index was 1.2 (1.12–1.28). The hazard ratio of subjects with high triglyceride and low high-density lipoprotein-cholesterol levels were 1.56 (1.12–2.18) and 1.52 (1.03–2.25), respectively.

**Conclusions:**

Obesity, high triglyceridemia, and low high-density lipoprotein-cholesterolemia are independent risk factors of alcoholic fatty liver disease in Japanese men who consumed alcohol habitually. In people with these risks, triglyceride lowering and high-density lipoprotein-cholesterol raising by improving insulin resistance and weight maintenance in addition to abstinence from alcohol would be effective in preventing the development of alcoholic fatty liver disease.

**Supplementary Information:**

The online version contains supplementary material available at 10.1186/s12876-021-01893-4.

## Background

As viral hepatitis is being eradicated, alcohol-related liver disease (ARLD) is attracting attention as a major cause of chronic liver disease, cirrhosis, as well as hepatocellular carcinoma [1, 2]. ARLD, is a chronic liver disorder caused by habitual consumption of alcohol [3]. For this reason, warnings and social support for alcohol dependence have been continuously implemented. However, the prevalence of ARLD is not decreasing, as it persists worldwide.

Simultaneously, hepatic steatosis has attracted attention in individuals who consume alcohol. Alcoholic fatty liver disease (AFLD) is a hepatic steatosis in individuals who consume alcohol habitually [4] and indeed a syndrome as a counter concept of non-alcoholic fatty liver disease (NAFLD) (Additional file 1: Figure S1). In NAFLD, hepatocyte steatosis due to overnutrition is considered the cause of cirrhosis and hepatocellular carcinoma [5, 6]. Furthermore, in viral hepatitis, it has been reported that steatosis caused by viral infection is a risk factor in the development of chronic fibrosis and hepatocellular carcinoma [7, 8]. Thus, there is a concern that hepatic steatosis itself may be a fundamental problem in various chronic liver diseases.

Historically, malnutrition has been the major problem in individuals with ARLD. On the other hand, epidemiological studies have shown that hepatic steatosis is a risk factor of cirrhosis in ARLD as well as NAFLD, and that cirrhosis is a risk factor in the development of hepatocellular carcinoma [9–11]. AFLD has been considered a risk factor of cirrhosis and hepatocellular carcinoma in addition to the volume of alcohol consumption or genetic susceptibility for them [12]. High-risk lifestyle including high fat diet and smoking, as well as body weight gain have been recognized as risk factors of cirrhosis and hepatocellular carcinoma, and also as underlying factors in individuals with AFLD [12]. These risk factors are also associated with metabolic syndrome. In fact, the prevalence of obesity and metabolic syndrome in ARLD patients is as high as 44% and 32%, respectively, and their comorbidity has been reported to be associated with increased liver-related mortality in ARLD patients [13]. Thus, metabolic syndrome might have a pivotal role in ARLD and NAFLD.

As described above, in ARLD, steatosis and metabolic syndrome are increasingly recognized as prognostic factors. However, compared with epidemiological studies on NAFLD, the role of metabolic syndrome in the natural history of AFLD has not been completely elucidated. Therefore, we performed a retrospective cohort study to determine the different influences of components of the metabolic syndrome on the development of AFLD.

## Methods

### Study population and design

In this study, we analyzed the data which was registered as the NAFLD in Gifu Area, Longitudinal Analysis (NAGALA) study. The NAGALA study database consists of the results of health check-up programs. Apparently healthy local citizens who live mainly in Gifu prefecture, the central region of Japan, received the health check-up programs at Asahi university hospital annually and biannually. The aim of the health check-up programs was to detect chronic diseases associated with lifestyle and malignant neoplasms in healthy citizens. Informed consent was obtained in the form of opt-out on the web-site (https://www.hosp.asahi-u.ac.jp/shinryo/rinsyokenkyu/). Subjects who were unwilling to participate were excluded from the study. The ethics committee of Asahi University Hospital approved the study (ID: 2018-09-01).

### Data collection and measurements

Anthropometric, clinical, and laboratory evaluation was conducted. Anthropometric data involved height, body weight, and blood pressure. Body mass index (BMI) was calculated as weight in kilograms divided by height in meters squared. A standardized self-administered questionnaire was used to collect information on each participant's medical history and lifestyle factors, including smoking habits, alcohol consumption, and physical activity. Smoking was defined as the use of tobacco and smoking status was categorized into three groups (never smoker, ex-smoker, and current smoker). We used data on the amount and type of alcoholic beverages consumed per week to estimate weekly ethanol intake. Subjects with an ethanol intake ≥ 420 g/week at baseline were defined as subjects who consume alcohol habitually [14]. Participants who regularly played some sport at least once a week were classified as exercisers.

Metabolic abnormality was defined as follows: (1) high blood pressure (systolic blood pressure ≥ 130 mmHg or diastolic blood pressure ≥ 85 mmHg or the use of oral antihypertensive medications); (2) high blood glucose (fasting blood glucose ≥ 100 mg/dl or receiving antidiabetic medications); (3) high triglycerides (fasting plasma triglycerides ≥ 150 mg/dl or receiving medications for high triglycerides); (4) low HDL (high-density lipoprotein) -cholesterol (fasting plasma HDL < 40 mg/dl or receiving medications for low HDL-cholesterol) [15].

### Definition of AFLD

AFLD was defined as fatty liver found in participants who consume alcohol habitually. Fatty liver was diagnosed by the findings of abdominal ultrasonography performed by a trained technician [16]. Among the four known diagnostic criteria (hepatorenal echo contrast, liver brightness, deep attenuation, and vascular blurring), hepatorenal echo contrast and liver brightness are required for fatty liver. The ultrasonographic definition is the same as those for NAFLD.

### Statistical analysis

Baseline characteristics were summarized as frequency (percentage) for categorical variables and as median [interquartile range (IQR)] for continuous variables. Characteristics at the onset of AFLD or at the time of the last physical examination of non-AFLD subjects were summarized in the same way. Comparisons of categorical variables between subjects with and without AFLD were performed with the Pearson's chi-squared test. Those of continuous variables were performed with the Mann–Whitney U test or Kruskal–Wallis test. Adjusted Cox proportional hazards models were used to estimate the adjusted hazard ratios (HRs) and 95% confidence intervals (CIs) for development of AFLD according to the components of the metabolic syndrome at baseline. Age, smoking status, and alcohol intake were considered as confounders. Statistical analysis was performed using JMP version 14.0.0 software (SAS Institute Inc., Cary, NC, USA). We used two-sided test, and *P* values of 0.05 or less were considered statistically significant.

## Results

**Fig. 1:**
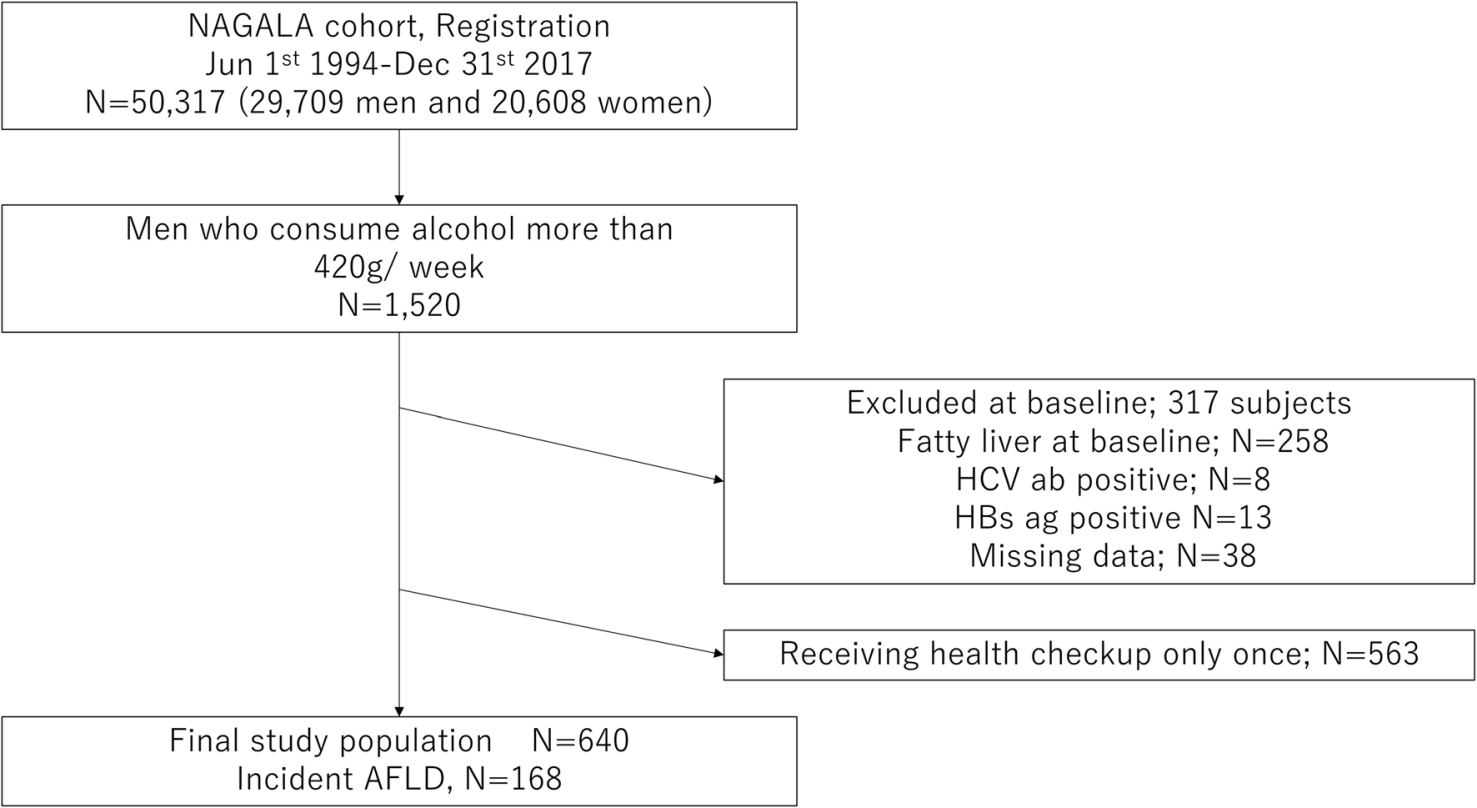
Flowchart of inclusion and exclusion criteria in men who consume alcohol more than 420 g/week. *NAGALA* NAFLD in Gifu area, longitudinal analysis, *AFLD* alcoholic fatty liver disease

The selection of subjects in the study is shown in Fig. 1. Of the 29,709 men who underwent physical examination at the health check-up center in Asahi University Hospital between 1994 and 2017, 1520 men with ethanol intake ≥ 420 g/week at the baseline examinations were included in this study. Among these subjects, 880 persons were excluded based on the following exclusion criteria: 258 had fatty liver at baseline; 21 had a positive serologic marker for hepatitis B surface antigen (HBsAg) or hepatitis C virus antibody (HCVAb) at baseline; missing data was found on 38 people; and 563 received health checkup only once. The total number of subjects who were eligible in the present study was 640.

From the 20,981 subjects whose alcohol intake was between 0 and 420 g/week, 11,014 subjects were excluded based on the same exclusion criteria and were additionally analysed. From the 6,185 men who did not consume any alcohol, 3602 men were excluded based on the same exclusion criteria and were additionally analysed (Additional file 2: Figure S2, Additional file 3: Figure S3).

Of the 640 subjects included in the longitudinal analysis, 168 subjects developed AFLD (Table 1). The median follow-up period was 3.91 years (IQR 1.63–7.09) and the incidence rate of AFLD was 49.1 cases per 1000 persons per year. Considering the baseline characteristics of subjects with and without incident AFLD, there were no differences between groups in age, habitual exercise, smoking states, presence of diabetes, and high blood glucose at baseline. The levels of fasting blood glucose, HbA1c, aspartate aminotransferase, gamma-glutamyl transferase, albumin, platelet, fibrosis-4 (FIB-4) index, NAFLD fibrosis score, and alcohol consumption were not different significantly among the two groups. In contrast, in those with incident AFLD, high blood pressure, high triglycerides, and low HDL-cholesterol accounted for 54.2%, 39.3%, and 23.2%, and in those without AFLD, 40.5%, 23.3%, and 10.4%, respectively, and significant differences were observed (*p* = 0.002, *p* < 0.0001, and *p* < 0.0001, respectively). BMI in the AFLD group was 23.6 kg/m^2^, while 22.0 kg/m^2^ in the no AFLD group, and a significant difference was observed (*p* < 0.0001). Total cholesterol, low-density lipoprotein (LDL)-cholesterol, and ALT also showed significant differences (*p* = 0.02, *p* = 0.019, and *p* = 0.012, respectively).

**Table 1:** Baseline characteristics of the study population

	No AFLD	Incident AFLD	*P* value
Number	472	168	
Exerciser, N (%)	93 (19.7)	24 (14.3)	0.12
Never smoker, N (%)	51 (10.8)	11 (6.5)	0.18
Ex smoker, N (%)	120 (25.4)	51 (30.4)	
Current smoker, N (%)	301 (63.7)	106 (63.1)	
Diabetes, N (%)	25 (5.3)	15 (8.9)	0.095
High blood pressure, N (%)	191 (40.5)	91 (54.2)	0.002
High blood glucose, N (%)	190 (40.3)	81 (48.2)	0.073
High triglycerides, N (%)	110 (23.3)	66 (39.3)	< 0.0001
Low HDL-cholesterol, N (%)	49 (10.4)	39 (23.2)	< 0.0001
Age, year	48 (14)	47 (14)	0.59
BMI, kg/m^2^	22 (3.1)	23.6 (3.8)	< 0.0001
Systolic blood pressure, mmHg	125 (19.5)	128 (19.9)	0.002
Diastolic blood pressure, mmHg	80 (14)	82 (13)	0.005
Fasting blood glucose, mg/dl	97 (13)	99 (15)	0.063
HbA1c, %	5.1 (0.6)	5.2 (0.8)	0.2
Total cholesterol, mg/dl	196 (49)	201 (45.5)	0.02
Triglyserides, mg/dl	98 (79.8)	132 (124.7)	< 0.0001
HDL-cholesterol, mg/dl	54.5 (21.5)	49.2 (20)	< 0.0001
LDL-cholesterol, mg/dl	112 (41.4)	117.3 (37.8)	0.019
Non HDL-cholesterol, mg/dl	137.3 (47.5)	147 (44.5)	< 0.0001
AST, IU/L	21(8)	22 (9)	0.87
ALT, IU/L	20 (11.7)	22 (12)	0.012
GGT, IU/L	38 (45.5)	42 (51.5)	0.078
Alb, g/dl	4.3 (0.3)	4.3 (0.3)	0.26
Platelet, 10^4^/mL	23.2 (6.7)	23.2 (8.1)	0.93
FIB4index	0.97 (0.54)	0.89 (0.46)	0.064
NAFLD Fibrosis score	− 2.55 (1.44)	− 2.46 (1.53)	0.8
Alcohol consumption, g/week	490 (154)	492 (154)	0.16

Data are expressed as frequency (percentages %) for categorical variables, or median (interquartile range) for continuous variables. Comparisons between the groups were performed with the Pearson's chi-square test for categorical variables and with the Mann–Whitney U test or Kruskal–Wallis test for continuous variables

*HDL* high-density lipoprotein, *LDL* low-density lipoprotein, *BMI* body mass index, *HbA1c* hemoglobin A1c, *AST* aspartate-aminotransferase, *ALT* alanine aminotransferase, *GGT* γ-glutamyltransferase, *Alb* albumin

We also compared the baseline characteristics of subjects with and without incident fatty liver in the subgroup with alcohol intake of less than 420 g/week. Of the 9,967 subjects with alcohol intake between 0 and 420 g/week, incident fatty liver was observed in 2465 subjects (Additional file 5: Table S1). The percentage of high blood glucose was 38.3% in subjects with incident fatty liver and 30.7% in those without incident fatty liver (*p* < 0.0001). There was no difference in alcohol consumption between the two groups. Of the 2583 subjects who did not consume alcohol, incident fatty liver was observed in 771 subjects (Additional file 6: Table S2). The percentage of high blood glucose was 35.5% in subjects with incident fatty liver and 26.9% in those without incident fatty liver (*p* < 0.0001).

Following baseline analysis, we compared the characteristics at the onset in subjects who had incident AFLD to the last characteristics of subjects without AFLD (Table 2). In subjects with incident AFLD, high blood pressure, high blood glucose, high triglycerides, and low HDL-cholesterol accounted for 64.3%, 61.9%, 45.2%, and 22.6%, respectively, and were significantly higher than those in subjects without AFLD of 49.6%, 48.7%, 20.8%, and 8.9%, respectively. BMI and body weight gain in the incident AFLD group were 24.2 kg/m^2^ and 1.5 kg, while 22.3 kg/m^2^ and 0.1 kg in the no AFLD group. The volume of habitual alcohol consumption in subjects with incident AFLD (462 g/week) was slightly higher compared to those without AFLD (371 g/week), but this was not significant.

**Table 2:** Characteristics at the onset of AFLD or at the last visit of non-AFLD subjects

	No AFLD	Incident AFLD	*P* value
Number	472	168	
Habitual alcohol consumption, N (%)	209 (44.3)	91 (54.2)	0.027
Exerciser, N (%)	110 (23.3)	24 (14.3)	0.013
Never smoker, N (%)	47 (9.9)	9 (5.4)	0.028
Ex smoker, N (%)	173 (36.7)	79 (47)	
Current smoker, N (%)	252 (53.4)	80 (47.6)	
Diabetes, N (%)	28 (5.9)	23 (13.7)	0.0014
High blood pressure, N (%)	234 (49.6)	108 (64.3)	0.001
High blood glucose, N(%)	230 (48.7)	104 (61.9)	0.0033
High triglycerides, N (%)	98 (20.8)	76 (45.2)	< 0.0001
Low HDL-cholesterol, N (%)	42 (8.9)	38 (22.6)	< 0.0001
Age, year	54 (13)	52 (13)	0.043
BMI, kg/m^2^	22.3 (3.5)	24.2 (3.8)	< 0.0001
Systolic blood pressure, mmHg	127 (20.4)	130 (14.5)	0.0035
Diastolic blood pressure, mmHg	80 (14.5)	83.8 (10.6)	0.0005
Fasting blood glucose, mg/dl	99 (13)	103 (15.8)	0.0003
HbA1c, %	5.2 (0.5)	5.4 (0.7)	0.024
Total cholesterol, mg/dl	198 (47)	204 (43)	0.0023
Triglyserides, mg/dl	85.5 (71)	136 (125.8)	< 0.0001
HDL-cholesterol, mg/dl	59 (23.7)	49.9 (19.7)	< 0.0001
LDL-cholesterol, mg/dl	112.8 (45.8)	119.2 (48.6)	0.027
Non HDL-cholesterol, mg/dl	136.7 (49.8)	153.9 (49)	< 0.0001
AST, IU/L	21 (9)	24 (13)	< 0.0001
ALT, IU/L	20( 11)	25 (17)	< 0.0001
GGT, IU/L	34.5 (36)	50 (59.7)	< 0.0001
Alb, g/dl	4.2 (0.3)	4.3 (0.3)	0.68
Platelet, 10^4^/mL	22.6 (6.8)	23.6 (8.1)	0.13
FIB4index	1.12 (0.7)	1.1 (0.68)	0.29
NAFLD Fibrosis score	− 2.12 (1.49)	− 2.19 (1.63)	0.90
Alcohol consumption, g/week	371 (244.7)	462 (310.9)	0.092
Body weight gain, kg	0.1 (3.6)	1.5 (3.5)	< 0.0001

Data are expressed as frequency (percentages %) for categorical variables, or median (interquartile range) for continuous variables. Comparisons between the groups were performed with the Pearson's chi-square test for categorical variables and with the Mann–Whitney U test or Kruskal–Wallis test for continuous variables

*HDL* high-density lipoprotein, *LDL* low-density lipoprotein, *BMI* body mass index, *HbA1c* hemoglobin A1c, *AST* aspartate aminotransferase, *ALT* alanine aminotransferase, *GGT* γ-glutamyltransferase, *Alb* albumin

Next, we evaluated the impact of each component of metabolic syndrome to incident AFLD using with Cox model (Table 3). After adjusting for age, smoking status, and alcohol consumption, the HR (95% CI) for a 1 kg/m^2^ increase in BMI was 1.2 (1.12–1.28, *p* < 0.0001). The HRs of subjects with high triglyceride and low HDL-cholesterol levels were 1.56 (1.12–2.18, *p* = 0.0093) and 1.52 (1.03–2.25, *p* = 0.034), respectively. Model 2, which was also adjusted for weight gain, showed a HR of 1.21 (1.14–1.29, *p* < 0.0001) for the development of AFLD with a 1 kg/m^2^ increase in BMI. The HRs of subjects with high triglyceride and low HDL-cholesterol levels were 1.71 (1.22–2.38, *p* = 0.0016) and 1.41 (0.96–2.07, *p* = 0.076), respectively.

**Table 3:** Adjusted Cox proportional hazards models for development of AFLD

	aHR (95% CI) ^a^	*P* value	aHR (95% CI) ^b^	*P* value
Incident AFLD	Model 1		Model 2	
Age (1 year)	1.01 (0.99–1.03)	0.18	1.03 (1.01–1.05)	0.0072
BMI (1 kg/m^2^)	1.2 (1.12–1.28)	< 0.0001	1.21 (1.14–1.29)	< 0.0001
High blood pressure	1.18 (0.85–1.65)	0.32	1.3 (0.93–1.82)	0.12
High blood glucose	1.04 (0.75–1.44)	0.8	1.11 (0.8–1.54)	0.53
High triglycerides	1.56 (1.12–2.18)	0.0093	1.71 (1.22–2.38)	0.0016
Low HDL cholesterol	1.52 (1.03–2.25)	0.034	1.41 (0.96–2.07)	0.076
Never smoker	Ref		Ref	
Ex smoker	1.6 (0.86–3.26)	0.14	1.38 (0.74–2.82)	0.32
Current smoker	1.61 (0.9–3.18)	0.11	1.28 (0.71–2.55)	0.42
Alcohol consumption (1 g/week)	1.00 (1.00–1.00)	0.98	1.00 (1.00–1.00)	0.91
Body weight gain (1 kg)			1.12 (1.08–1.17)	< 0.0001

*BMI* body mass index, *HDL* high-density lipoprotein, *HR* hazard ratio, *CI* confidence interval

^a^Data adjusted for age, BMI, high blood pressure, high blood glucose, high triglycerides, low HDL cholesterol, smoking status, alcohol consumption

^b^Data adjusted for age, BMI, high blood pressure, high blood glucose, high triglycerides, low HDL cholesterol, smoking status, alcohol consumption, body weight gain

We also evaluated the impact of each component on incident fatty liver in the subgroup with an alcohol intake of less than 420 g/week. In the group with alcohol intake between 0 and 420 g/week, after adjustment, the HR (95% CI) for a 1 kg/m^2^ increase in BMI was 1.2 (1.18–1.22, *p* < 0.0001) (Additional file 7: Table S3). The HRs of subjects with high triglyceride, low HDL-cholesterol, and high glucose levels were 1.71 (1.55–1.89, *p* < 0.0001), 1.17 (1.06–1.29, *p* = 0.002), and 1.34 (1.23–1.46, *p* < 0.0001), respectively. In the group that consumed no alcohol, after adjustment, the HR (95% CI) for a 1 kg/m^2^ increase in BMI was 1.21 (1.17–1.24, *p* < 0.0001) (Additional file 8: Table S4). The HRs of subjects with high triglyceride, low HDL-cholesterol, and high glucose levels were 1.26 (1.04–1.51, *p* = 0.0167), 1.34 (1.15–1.57, *p* = 0.0003), and 1.43 (1.23–1.68, *p* < 0.0001), respectively.

## Discussion

The incidence rate of AFLD was 49.1 cases per 1000 persons per year. Obesity, high triglyceridemia, and low HDL-cholesterolemia were independent risk factors of AFLD in Japanese men who consume alcohol. On the other hand, among the components of the metabolic syndrome, hypertension and high glucose were not significant risk factors. In addition to AFLD risk factors, high glucose was a significant risk factor for incident fatty liver in subjects whose alcohol consumption was less than 420 g/week.

Fatty liver in non-drinking citizens is widely recognized as NAFLD. NAFLD is the hepatic phenotype of the metabolic syndrome, and obesity and metabolic abnormalities are risk factors for its incidence [17]. Nonalcoholic steatohepatitis (NASH) is observed in a part of individuals with NAFLD, and the pathology of NASH seems to be similar to that of chronic hepatitis in subjects with ARLD [18]. NASH is a risk factor of liver cirrhosis and hepatocellular carcinoma [19, 20]. These findings suggest that hepatic steatosis in subjects who consume alcohol habitually, in other words AFLD, could be a risk factor of liver cirrhosis and hepatocellular carcinoma [1, 2]. In addition to that, hyperglycemia co-exists with AFLD. We previously reported that the prevalence of hyperglycemia was high in subjects with AFLD [21]. However, the causal relationship of hyperglycemia with AFLD has not been clarified yet. While all components of metabolic syndrome are strong risk factors of incident NAFLD [15, 22], our current study revealed that three of the five components of the metabolic syndrome, obesity, hypertriglyceridemia, and low HDL-cholesterolemia, are significant risk factors of incident AFLD. Previous report showed that overweight individuals are more likely to develop hypertriglyceridemia due to alcohol consumption than lean individuals [23], and individuals with obesity and abnormal lipid levels may synergistically induce liver steatosis under alcohol consumption. Abstinence from alcohol alone is not sufficient to prevent the development of AFLD, and attention to metabolic abnormalities is also necessary. Weight maintenance in obese alcohol consumers and improvement of insulin resistance in alcohol consumers with high triglycerides and low HDL-cholesterol levels may be important in preventing the development of fatty liver.

In patients with metabolic syndrome, chronic inflammation of visceral adipose tissue leads to increase in free fatty acid (FFA) from adipocytes. High level serum FFA increases hepatic FFA uptake, which subsequently upregulates very low-density lipoprotein (VLDL) synthesis in the liver. Upregulated hepatic FFA uptake also increased carbohydrate-derived de novo lipogenesis via increased sterol regulatory element-binding protein (SREBP)-1c expression [24]. Adipokine abnormalities, such as decreased adiponectin, play a role in fat accumulation [25].

On the other hand, alcohol consumption increases the hepatic nicotinamide adenine dinucleotide hydroxide/nicotinamide adenine dinucleotide ratio, which leads to impaired mitochondrial beta-oxidation of fatty acids, increased fatty acid synthesis, and increased hepatic triglycerides [26]. Alcohol can upregulate SREBP-1c expression directly via acetaldehyde and indirectly via stimulation of factors including early growth response protein-1 and tumor necrosis factor-α [27, 28]. Alcohol also downregulates factors that decrease SREBP-1c expression, including adiponectin [29]. Ethanol consumption directly or indirectly inhibits peroxisome proliferator-activated receptor-α [30, 31]. Furthermore, adenosine monophosphate-activated protein kinase (AMPK) increases acetyl coenzyme A carboxylase (ACC) activity and decreases carnitine palmitoyltransferase (CPT)-1 activity, resulting in fat accumulation [32]. Thus, NAFLD and AFLD share common etiological mechanisms, such as altered expression of lipid metabolism-related transcription factors and abnormalities in adipokines (Additional file 4: Figure S4). These mechanisms can explain the similarity in pathological features and natural history. Hypertension and hyperglycemia, rather than obesity and abnormal lipid metabolism, may be the unique pathogenetic factors in NAFLD, but these need to be assessed by future research.

In our study, alcohol intake at baseline did not predict the incidence of AFLD in subjects who consume alcohol habitually, nor did it predict the incidence of fatty liver in subjects whose alcohol consumption was less than 420 g/week. In individuals who consume alcohol habitually, the amount of alcohol is known to be a risk factor for the incidence of chronic liver disease [33, 34]. On the other hand, no study has directly demonstrated that the amount of alcohol is a risk factor for the incidence of AFLD in individuals who consume alcohol habitually. It has also not been shown that the incidence of fatty liver increases along with the amount of alcohol intake in individuals who consume alcohol habitually. Although not a direct study of the incident of AFLD in individuals who consume alcohol habitually, an Italian study of the general population showed that alcohol intake was not associated with fatty liver [35]. This result is consistent with our study.

A lipid profile of high triglycerides and low HDL-cholesterol is common in subjects with fatty liver [36]. Insulin resistance is a strong underlying mechanism of this dyslipidemia [37], leading to increased rates of hepatic triglyceride synthesis and VLDL particle production, followed by low HDL-cholesterol [38]. While alcohol consumption does not produce favorable changes in hypertriglyceridemia, it may induce an increase in HDL levels by a mechanism that inhibits cholesteryl ester transfer protein (CETP) activity [39, 40]. Although HDL plays beneficial multifaceted roles, in the presence of inflammation, oxidative stress, and glycemic abnormalities, HDL particles may be converted to dysfunctional molecules with proatherogenic effects [41, 42]. Circulating levels of HDL2 are reduced in patients with NAFLD, suggesting the presence of dysfunctional HDL particles in these patients [43]. The protective effect of high HDL-cholesterolemia against the development of AFLD and the importance of HDL quality in AFLD patients have not yet been investigated, and further studies are needed.

Our study has limitations. First, our database consisted of apparently healthy citizens who underwent a health check-up program. Our sample may contain more health-conscious people than general population. While, our epidemiological study is complementary to epidemiological studies that consist of hospital patients who consume alcohol habitually. Second, this study was conducted in Japanese men, and the results may differ in other racial and ethnic groups.

## Conclusions

Our study shows that obesity, high triglyceridemia, and low HDL-cholesterolemia are independent risk factors of incident AFLD in Japanese men who consume alcohol habitually. In patients with these risks, triglyceride lowering and HDL-cholesterol raising by improving insulin resistance and weight maintenance in addition to abstinence from alcohol would be effective in preventing the development of AFLD.

## Supplementary Information

**Additional file 1**. **Figure S1**. Relationship between fatty liver and ARLD. ARLD is described as chronic liver disease caused by excessive alcohol use. Fatty liver in excessive alcohol users is called AFLD and is a part of ARLD. Excessive alcohol use in men is defined as consumption of more than 420 g/week. On the other hand, fatty liver in low drinkers is known as NAFLD. Low alcohol consumption in men is defined as consumption of less than 210 g/week. AFLD, alcoholic fatty liver disease; ARLD, alcohol-related liver disease; FLD, fatty liver disease; NAFLD, non-alcoholic fatty liver disease.

**Additional file 2**. **Figure S2**. Flowchart of inclusion and exclusion criteria in men who consume alcohol more than 0 g and less than 420 g/week. NAGALA, NAFLD in Gifu area, longitudinal analysis.

**Additional file 3**. **Figure S3**. Flowchart of inclusion and exclusion criteria in men who consume no alcohol. NAGALA, NAFLD in Gifu area, longitudinal analysis.

**Additional file 4**. **Figure S4**. Mechanisms of hepatic steatosis. The common etiological mechanisms shared by NAFLD and AFLD is expressed in this figure. AMPK, AMP-activated protein kinase; FFA, free fatty acid; NAD, nicotinamide adenine dinucleotide; NADH, nicotinamide adenine dinucleotide hydroxide; PPAR, peroxisome proliferator-activated receptor; SREBP, sterol regulatory element-binding protein.Figure S4. Mechanisms of hepatic steatosis. The common etiological mechanisms shared by NAFLD and AFLD is expressed in this figure. AMPK, AMP-activated protein kinase; FFA, free fatty acid; NAD, nicotinamide adenine dinucleotide; NADH, nicotinamide adenine dinucleotide hydroxide; PPAR, peroxisome proliferator-activated receptor; SREBP, sterol regulatory element-binding protein.

**Additional file 5**. **Table S1**. Comparison of subjects who consume alcohol more than 0 g and less than 420 g/week with and without fatty liver. Data are expressed as frequency (percentages %) for categorical variables, or median (interquartile range) for continuous variables. Comparisons between the groups were performed with the Pearson's chi-square test for categorical variables and with the Mann-Whitney U test or Kruskal-Wallis test for continuous variables. HDL, high-density lipoprotein; LDL, low-density lipoprotein; BMI, body mass index; HbA1c, hemoglobin A1c; AST, aspartate-aminotransferase; ALT, alanine aminotransferase; GGT, γ-glutamyltransferase; Alb, albumin.

**Additional file 6**. **Table S2**. Comparison of subjects who consume no alcohol with and without fatty liver. Data are expressed as frequency (percentages %) for categorical variables, or median (interquartile range) for continuous variables. Comparisons between the groups were performed with the Pearson's chi-square test for categorical variables and with the Mann-Whitney U test or Kruskal-Wallis test for continuous variables. HDL, high-density lipoprotein; LDL, low-density lipoprotein; BMI, body mass index; HbA1c, hemoglobin A1c; AST, aspartate-aminotransferase; ALT, alanine aminotransferase; GGT, γ-glutamyltransferase; Alb, albumin.

**Additional file 7**. **Table S3**. Adjusted Cox proportional hazards models for developing fatty liver in subjects with alcohol intake more than 0 g and less than 420 g/week. ^a^Data adjusted for age, BMI, high blood pressure, high blood glucose, high triglycerides, low HDL cholesterol, smoking status, alcohol consumption. BMI, body mass index; HDL, high-density lipoprotein; HR, hazard ratio; CI, confidence interval.

**Additional file 8**. **Table S4**. Adjusted Cox proportional hazards models for developing fatty liver in subjects who consume no alcohol. ^a^Data adjusted for age, BMI, high blood pressure, high blood glucose, high triglycerides, low HDL cholesterol, smoking status, alcohol consumption. BMI, body mass index; HDL, high-density lipoprotein; HR, hazard ratio; CI, confidence interval.

## Data Availability

The datasets used and/or analysed during the current study are available from the corresponding author on reasonable request.

## Abbreviations

ACC: Acetyl coenzyme A carboxylase
AFLD: Alcoholic fatty liver disease
ALT: Alanine aminotransferase
AMPK: AMP-activated protein kinase
ARLD: Alcohol-related liver disease
AST: Aspartate aminotransferase
BMI: Body mass index
CETP: Cholesteryl ester transfer protein
CI: Confidence interval
CPT: Carnitine palmitoyltransferase
FFA: Free fatty acid
GGT: γ-Glutamyltransferase
HbA1c: Hemoglobin A1c
HBsAg: Hepatitis B surface antigen
HCVAb: Hepatitis C virus antibody
HDL: High-density lipoprotein
HR: Hazard ratio
IQR: Interquartile range
LDL: Low-density lipoprotein
NAFLD: Non-alcoholic fatty liver disease
NAGALA: NAfld in Gifu area, longitudinal analysis
NASH: Nonalcoholic steatohepatitis
SREBP: Sterol regulatory element-binding protein
VLDL: Very low-density lipoprotein
