# Supplementary figures and images for: Obesity and metabolic abnormalities as risks of alcoholic fatty liver in men: NAGALA study
Source: BMC Gastroenterol. 2021 Aug 9;21:321. doi: 10.1186/s12876-021-01893-4 (PMC8353849; doi:10.1186/s12876-021-01893-4)

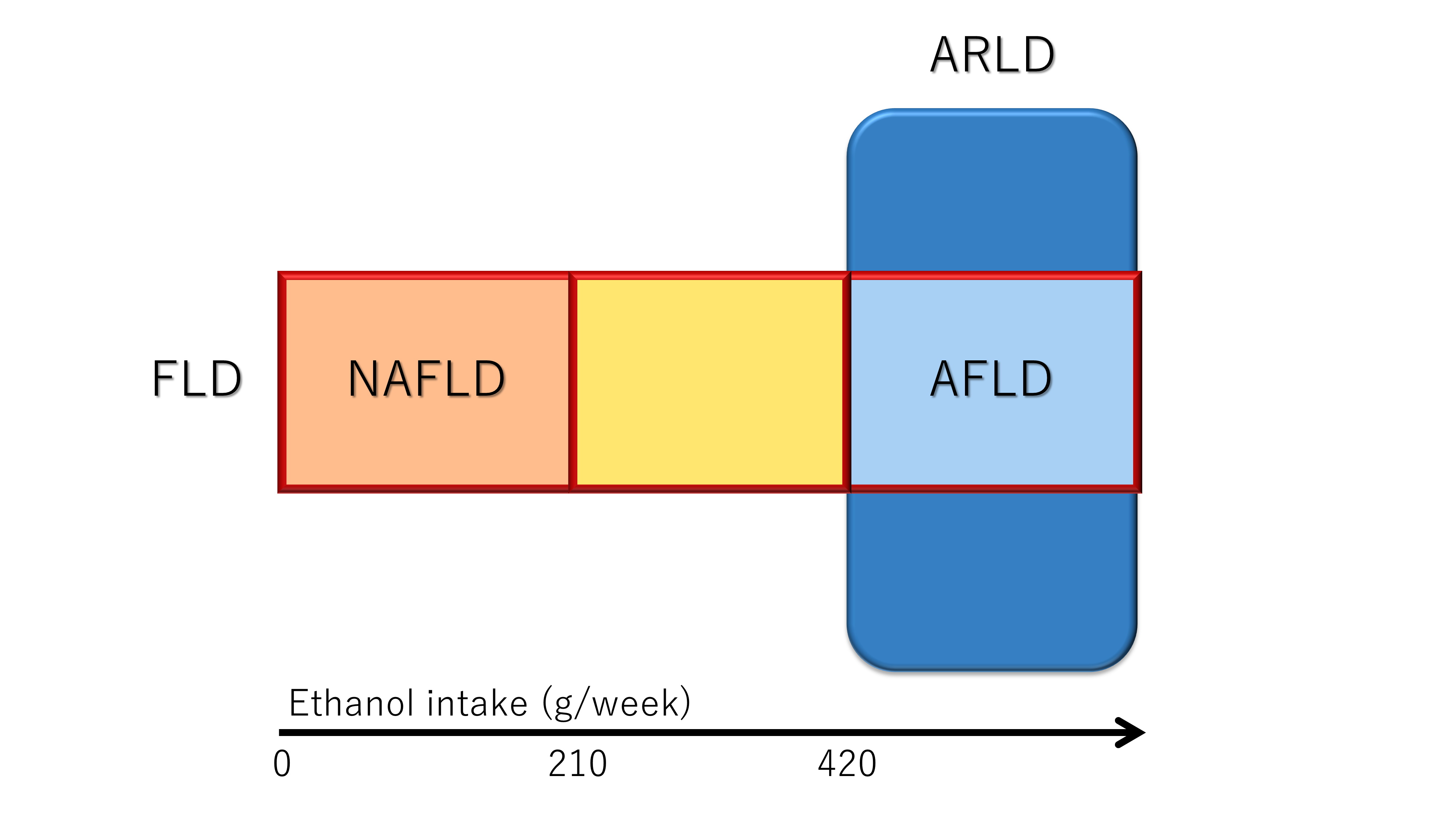

Supplement: Supplementary file 1 — Additional file 1. Figure S1. Relationship between fatty liver and ARLD. ARLD is described as chronic liver disease caused by excessive alcohol use. Fatty liver in excessive alcohol users is called AFLD and is a part of ARLD. Excessive alcohol use in men is defined as consumption of more than 420 g/week. On the other hand, fatty liver in low drinkers is known as NAFLD. Low alcohol consumption in men is defined as consumption of less than 210 g/week. AFLD, alcoholic fatty liver disease; ARLD, alcohol-related liver disease; FLD, fatty liver disease; NAFLD, non-alcoholic fatty liver disease. [file 12876_2021_1893_MOESM1_ESM.tif]

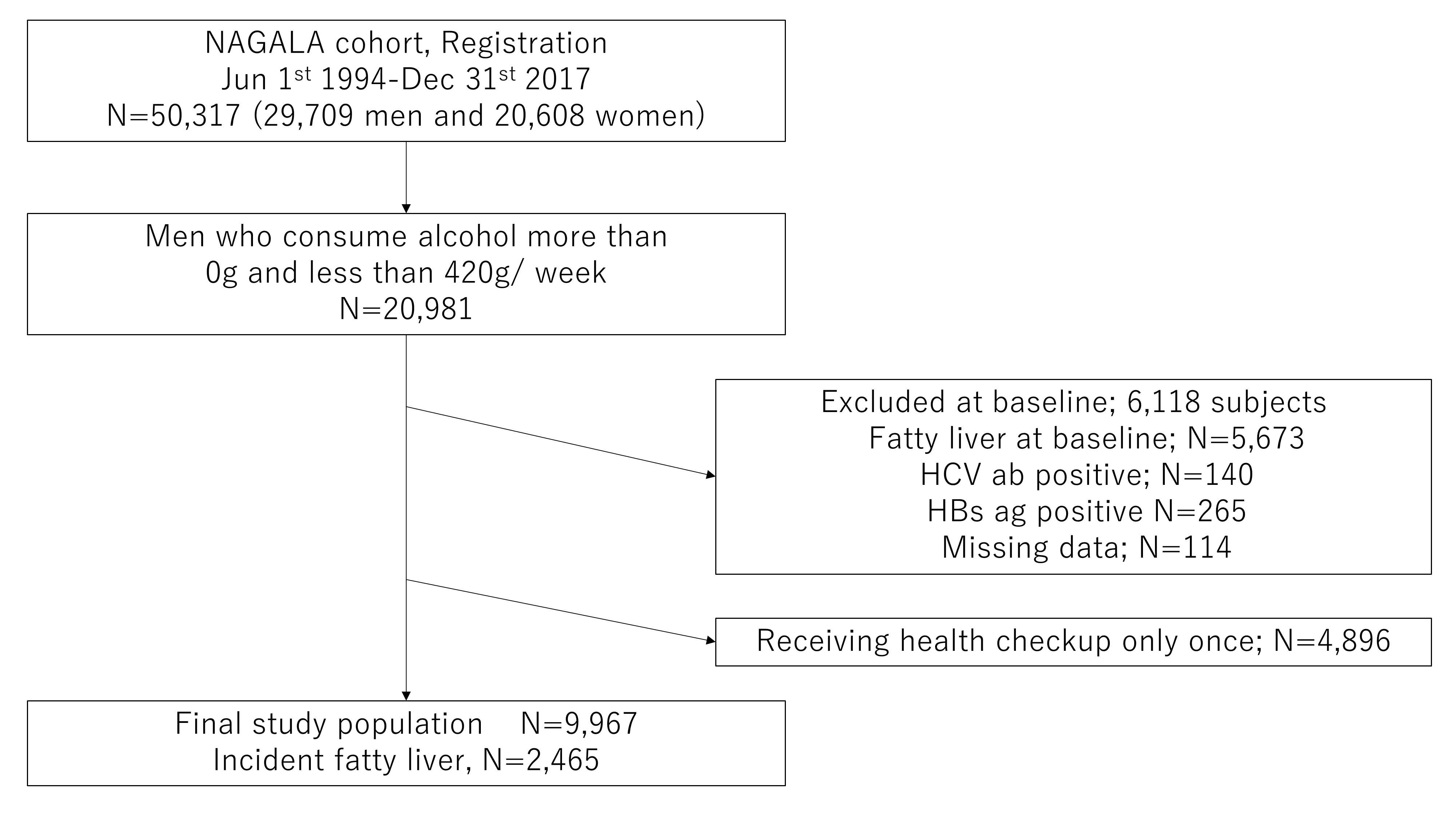

Supplement: Supplementary file 2 — Additional file 2. Figure S2. Flowchart of inclusion and exclusion criteria in men who consume alcohol more than 0 g and less than 420 g/week. NAGALA, NAFLD in Gifu area, longitudinal analysis. [file 12876_2021_1893_MOESM2_ESM.tif]

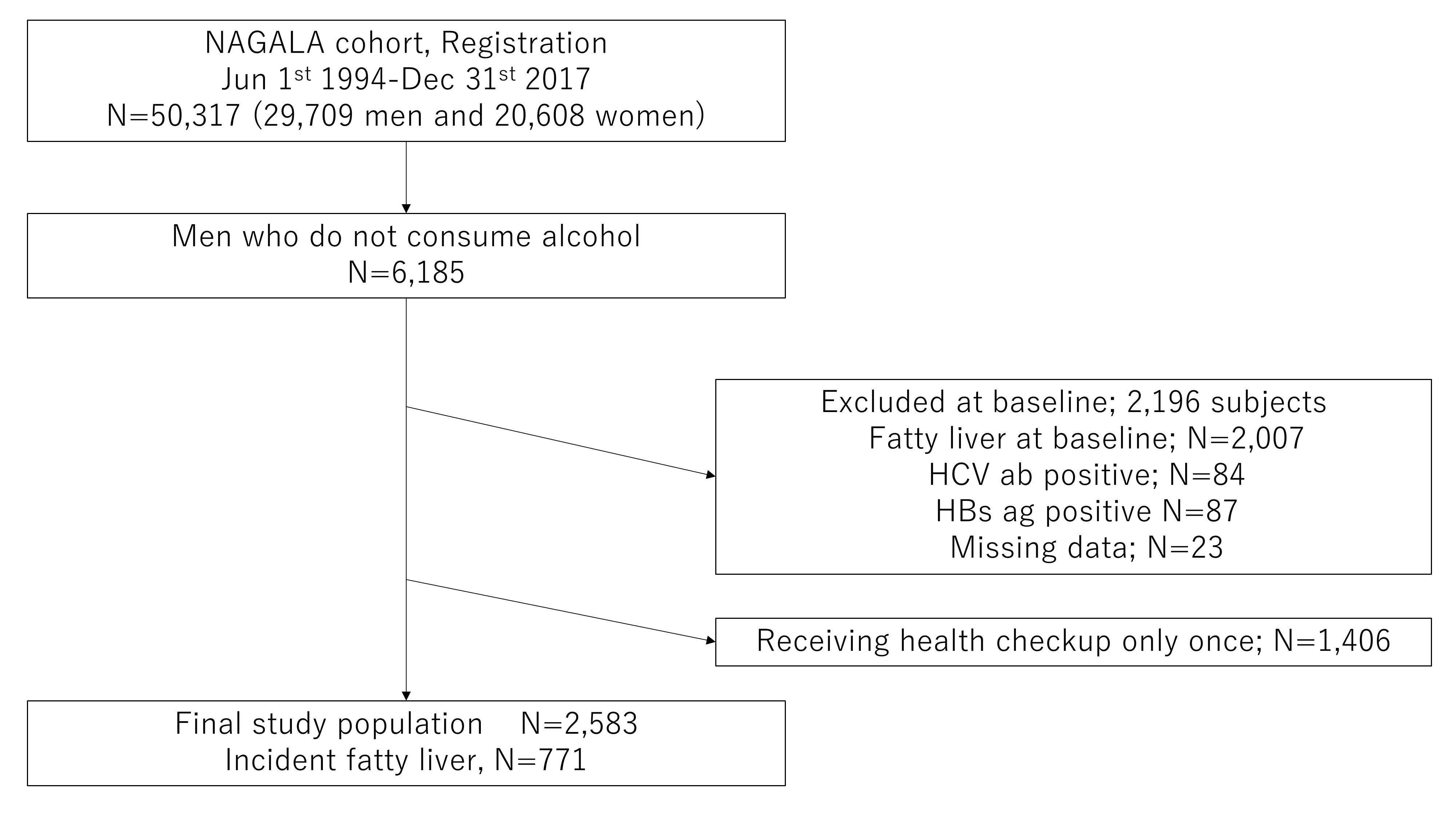

Supplement: Supplementary file 3 — Additional file 3. Figure S3. Flowchart of inclusion and exclusion criteria in men who consume no alcohol. NAGALA, NAFLD in Gifu area, longitudinal analysis. [file 12876_2021_1893_MOESM3_ESM.tif]

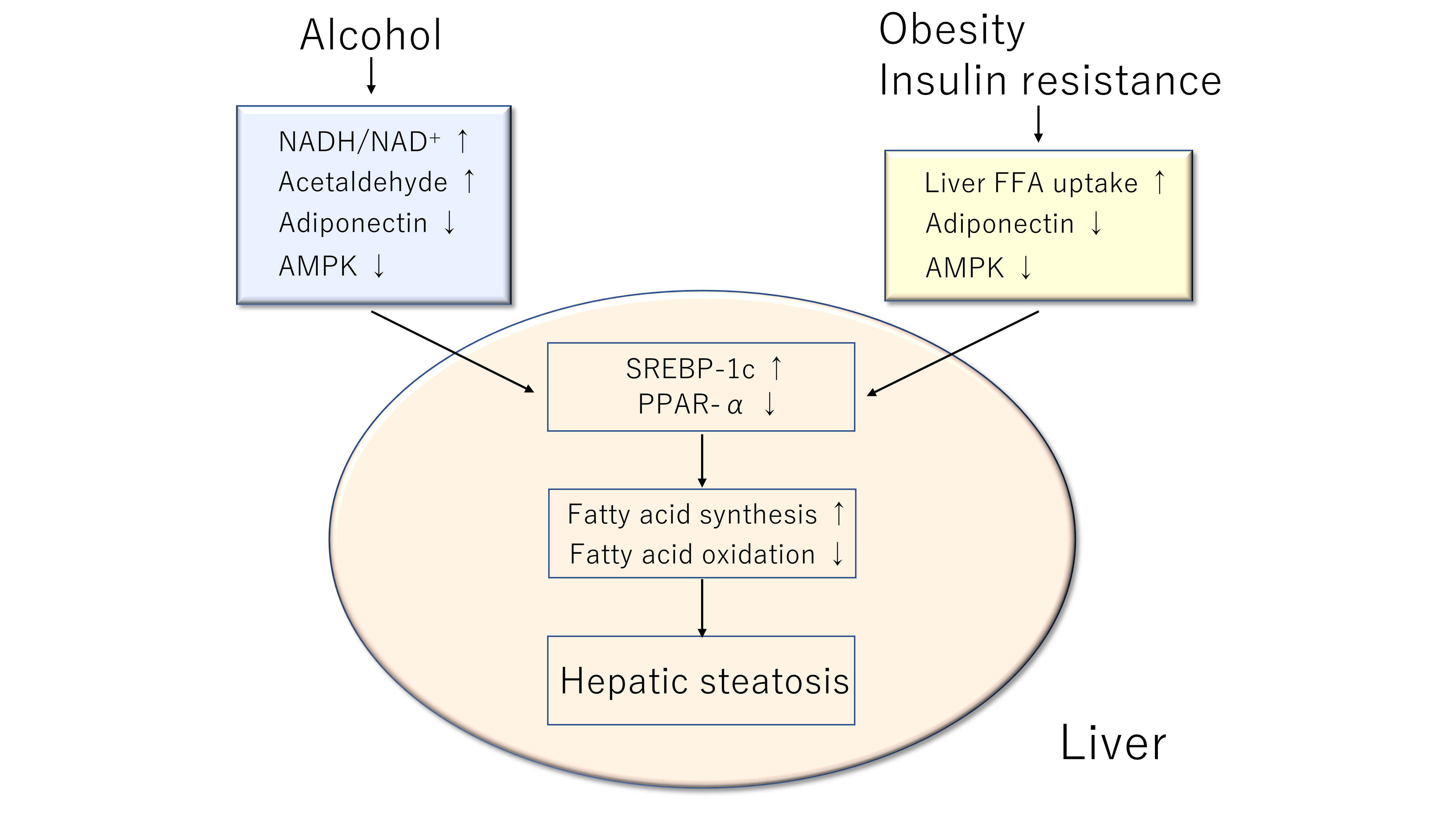

Supplement: Supplementary file 4 — Additional file 4. Figure S4. Mechanisms of hepatic steatosis. The common etiological mechanisms shared by NAFLD and AFLD is expressed in this figure. AMPK, AMP-activated protein kinase; FFA, free fatty acid; NAD, nicotinamide adenine dinucleotide; NADH, nicotinamide adenine dinucleotide hydroxide; PPAR, peroxisome proliferator-activated receptor; SREBP, sterol regulatory element-binding protein.Figure S4. Mechanisms of hepatic steatosis. The common etiological mechanisms shared by NAFLD and AFLD is expressed in this figure. AMPK, AMP-activated protein kinase; FFA, free fatty acid; NAD, nicotinamide adenine dinucleotide; NADH, nicotinamide adenine dinucleotide hydroxide; PPAR, peroxisome proliferator-activated receptor; SREBP, sterol regulatory element-binding protein. [file 12876_2021_1893_MOESM4_ESM.tif]
